# Supplementary material for: Identification and DNA Marker Development for a Wheat-Leymus mollis 2Ns (2D) Disomic Chromosome Substitution
Source: Int J Mol Sci. 2022 Feb 28;23(5):2676. doi: 10.3390/ijms23052676 (PMC8911044; doi:10.3390/ijms23052676)
Supplement: Supplementary file 1 [file ijms-23-02676-s001.zip › ijms-1548534-supplementary.pdf]

Supplementary Table S1 Genotype data obtained using wheat 55K SNP arrays for 17DM48 and its parents.

Chr	No. of makers	No. of valid makers in 7182	No. of valid makers in *P. huashanica*	No. of valid makers in 17DM48	No. of same makers (17DM48 vs 7182)	Percentage of same makers (17DM48 vs 7182)	No. of same makers (17DM48 vs *P. huashanica*)	Percentage of same makers (17DM48 vs *P. huashanica*)
1A	2624	2567	1503	2528	1154	43.98%	240	9.15%
1B	2595	2530	1523	2525	963	37.11%	252	9.71%
1D	2138	2122	1224	2119	1622	75.87%	30	1.40%
2A	2622	2586	1601	2586	729	27.80%	355	13.54%
2B	2600	2517	1491	2501	1353	52.04%	163	6.27%
2D	2247	2185	1302	836	278	12.37%	652	29.02%
3A	2174	2123	1229	2101	1125	51.75%	156	7.18%
3B	2595	2536	1530	2525	1446	55.72%	150	5.78%
3D	1693	1679	953	1669	1242	73.36%	44	2.60%
4A	2592	2556	1465	2553	1393	53.74%	147	5.67%
4B	2556	2520	1509	2528	1788	69.95%	62	2.43%
4D	1420	1406	854	1410	1050	73.94%	17	1.20%
5A	2611	2568	1456	2555	1588	60.82%	133	5.09%
5B	2586	2537	1499	2523	961	37.16%	227	8.78%
5D	1737	1718	931	1725	1258	72.42%	36	2.07%
6A	2623	2562	1540	2533	1563	59.59%	146	5.57%
6B	2547	2456	1506	2482	1625	63.80%	78	3.06%
6D	1728	1696	968	1709	715	41.38%	164	9.49%
7A	2579	2479	1534	2515	1529	59.29%	138	5.35%
7B	2487	2430	1450	2399	1282	51.55%	205	8.24%
7D	2305	2290	1293	2278	1639	71.11%	57	2.47%
total	49059	48063	28361	46600	26303	53.62%	3452	7.04%

Supplementary Table S2 PLUG and EST–STS markers used for the identification of alien chromosomes for 17DM48.

Marker	Type	Primer	Location	Gel type/restriction enzyme	Tm °C/t (h)
BG607805	EST-STS	F:ACCCCTTAGAGACGCTCCAT<br>R:TAGCTCTTTGCTTGCCCAAT	2AL 2AS 2BS	8% non-denaturing polyacrylamide gel/-	58 /-
CD453246	EST-STS	F:GAGAGATCTCGTCCAGCGAC<br>R:GGTCCCAACTTCGTCTGGT	2AS 2BS 2DS	8% non-denaturing polyacrylamide gel/-	64 /-
BQ169707	EST-STS	F:AATCAATCAATGGACGGACG<br>R:GAGATGTTGACGCGGAAGAG	2AS 2BS 2DS	8% non-denaturing polyacrylamide gel/-	64 /-
TANC1139	PLUG	F:ATGTTGTCCATGCCTCCACTT<br>R:CTGGAATTCTCCGTCTGCTTA	2AL 2BL 2DL	2% agarose gel/TaqI/HaeIII	60/2 or 37/3
TANC1204	PLUG	F:GAGAGGAATGCGTGAAGTTTG<br>R:AGACCATCTTTCCGGTCTTTG	2AL 2BL 2DL	2% agarose gel/TaqI/HaeIII	60/2 or 37/3
TANC1210	PLUG	F:TTGTGACTGACAGCAACATCC<br>R:AGAGCTTGGCCTTCTCTTCC	2AL 2BL 2DL	2% agarose gel/TaqI/HaeIII	60/2 or 37/3

Supplementary Table S3 All SLAF-based markers designed for 17DM48.

Marker	Tm(◦C)	Primer(5′-3′)
LM51499	56	F: CAGCAGTGGCTTCTGTTCC<br>R: TGTATGTGCGGGAGTGGA
LM224473	54	F: GGACGGTGAGCAAGAAGG<br>R: CGTAATGCCCACGAAACA
LM134368	52	F: TTCATCCTGTTGTCCCTG<br>R: ATGGTGCTAAGCATTTCC
LM72199	52	F: CAAGGAAACGACGCAAGT<br>R: ATGTGAACAAAGCGGGAT
LM152390	52	F: TTTCTAGCCGCTAAAGGT<br>R: TTTCCAAGCCTACTCCTG
LM38697	58	F: CACAAGGAGAAGGCGGACTG<br>R: GTTGGGCTGGCTGATGCT
LM174359	54	F: GGGACCGACCATTGTTTA<br>R: CTTCCTCATCTGCCTGCT
LM139972	52	F: TTCATCCTGTTGTCCCTG<br>R: ATGGTGCTAAGCATTTCC
LM123894	56	F: AGGATTTCCATCTGGGTTGC<br>R: CGGGATGCCATTGTGCTT
LM14836	54	F: CCAAGAGCAGCAGCAAAC<br>R: GCATGACGAAGTCCCATA
LM25058	54	F: AGGAAGGGTCGGAAACTC<br>R: AACACCACGGAATGAAGC
LM36645	56	F: AACCATCCGCACATTAGAG<br>R: ACTGCCAATCAGAACCATAC
LM40281	54	F: ACATCCGAGCAAGAGCAG<br>R: CAAAGAACCACCGAAACC
LM40993	52	F: TTGTTCCACTTATCCTCCTC<br>R: ATTGCCTGCTTCTTCTCA
LM46183	52	F: CCTTCGTGTATCGCTTTC<br>R: CCATAATTTGCACCAACC
LM56467	58	F: GCTACAACCGCTCCCTTTA<br>R: TACCCAGAAGAACAAGTACACC
LM56721	58	F: TCTCGACATGGTCCACAAC<br>R: GGTCTATTGGTACAGGGTGC
LM61924	52	F: AAACCGCAAGCACAAGAA<br>R: GATGAACCCTACAGTCACCAC
LM11028	54	F: TACCGATGGTGATGATGG<br>R: TGATGTTGTTGCTGTCCC
LM13006	56	F: TGCGGTTGCGTCTATTGG<br>R: TGCTGGTGCATCATCATCG
LM14528	56	F: CCTCCGCTTGACACGATA<br>R: CAGCCTGCGATGTTGGTA
LM15560	54	F: CGATACCGCAGGGATAGA<br>R: GCAACCCAACAGCAAGAT
LM17228	56	F: GCTCCTTTCTCGCTTGCT<br>R: TGGACCGCTACGTTTGAC
LM19428	56	F: CGTCATCCTCCACCACCT<br>R: ACGCAATCTGCTCAACCC
LM19474	52	F: TCGTCTGGGTTTGCTTAT<br>R: CACCGATTTCCAAGTTTC
LM15952	56	F: TGGTAGGCAGACTTGGTG<br>R: TGTAAGGGCTGATGGGAC
LM19899	56	F: CGCCGCATCACTTCATTG

		R: AACCGCTCGCCGTTACTC
LM20199	56	F: TCCACCATTCTTCACCATC<br>R: GCATCAGTTCCCAACCATA
LM22331	54	F: TGGGAATGCCTACTGATG<br>R: CTGCTGGCTAAACAAGGT
LM24196	54	F: AACATCACGAGCGAGGAA<br>R: AACATCACGAGCGAGGAA
LM26048	56	F: TTTGGACTGGCTATGAGGG<br>R: GTCGGGACACGGGATTTA
LM24864	56	F: CTTGAAGAACAGGTGGGC<br>R: CTCGGAATCAGCTAGGAATT
LM27891	56	F: GTCGCCTCTTGTTCCCTG<br>R: CGTCGCCGATTCTCCTAA
LM28554	54	F: CTGCCACTTTGTGAAGGA<br>R: TAGCGTATCGTCACCTCC
LM30478	56	F: TGTCGTTGGTTCCCTCTAA<br>R: GTTTGCTGGTTTCTTGTCG
LM33500	56	F: CCGACGATGATGTAGTGAG<br>R: GACTTGGGAAGGAAGAAGA
LM27215	56	F: CAACCAAACTGACCGAGAC<br>R: AGGAAACGGCAGGAGAAG
LM33514	54	F: CGATTGTTCCGTTGGTGT<br>R: CTGACGTATGCCGGAGTT
LM37347	56	F: GCTTCTTCTTCGCAGTCG<br>R: CAGGGAGGTCAAGGGTGT
LM38912	56	F: ACAATGTGGAGCGGTGTC<br>R: GATGTGGCTGGCTGCTAT
LM45116	54	F: CGTTCTCCACCGTGTTAT<br>R: TACTGGGAGGCAACTGAT
LM45259	62	F: TTCTCCCTCCAGGTCCAAAG<br>R: TCTAAAGTGGCAGTCCCAACC
LM41719	54	F: CCTCATGCTGCATTGACC<br>R: GCATCGCCTGAAGGTTTT
LM42164	52	F: GTGGCGAAATGAATGTGC<br>R: TTACGCTTGGCTTGTTTG
LM48964	52	F: TCAACAAATCAGCCCACT<br>R: TTCTTCCCTGTTTCATCG
LM46500	52	F: ACGGAATCAATGAAAGCG<br>R: CCAAAGAATGTGGGAGGA
LM52860	54	F: TCTCCTGTGCTGCTTCTT<br>R: GTATGCGAACTGTGGTGC
LM50788	52	F: ATTTAGAGCCAATCGTGG<br>R: AAGGACATGGGAGAAGCA
LM51349	54	F: GTCCTCCTTCAACAACGG<br>R: CACCCAAGTGTCATCCAA
LM51704	54	F: CAGAAAGCACGAACCCAA<br>R: TCCGACCAGAAAGAGCAA
LM56049	56	F: CCAAACACCGAGAAACCG<br>R: GGACCAGCGAAGACAAGC
LM57217	56	F: GCGTCATCATTGTCACCG<br>R: CTCCTGTTGCCGCCTACT
LM63584	56	F: CCCATTAGTAAGGCACCG<br>R: CTCCCATCCAGATCACCC
LM68481	52	F: TTTATGTGGCTTGCTGTG<br>R: ATGCTCGTCTGTTGGTGT
LM65677	54	F: CAGAGCATAACCCAGGAG<br>R: CCATAGGAACAAGCCAGA

LM73267	56	F: GTTTGGCATCAGGTTCCG<br>R: GCTTGGTTGTGACCAGCAT
LM14172	54	F: TTCTGCCTCAAGACAAACA<br>R: GTTGACGAACCACGCTTA
LM34623	56	F: CCAGGTAGGTAACGAGTTT<br>R: GCAGCGATCTTGAGGTGT
LM21531	54	F: CGGAAATAAAGAGGCAAGT<br>R: TCACCAGATCCGTATCGTC
LM15193	54	F: ATTTGGCAGGTGGAAACG<br>R: CTCTGTTGGGCTGGCTTA
LM7529	52	F: AGGTTTCCAAATAAGGGAT<br>R: CGGACCGTGAATACTCTG
LM17738	54	F: ACCAACGCATCAAATCCC<br>R: CGCCAGTAGAGGTGGAGATA
LM23891	56	F: TGGGCAACCGATGCTCTA<br>R: ACTGGCACGAATCCGTCT
LM5642	58	F: GGATGGCTGGTAGGCGAGAA<br>R: CCGCGAACAGGAACACGA
LM29058	54	F: TAGGGCATTGTCGGTGTA<br>R: CGCAGGCTAATAGGTAAAA
LM19993	56	F: TCACCTCTTGACCACCCT<br>R: TGAACCCTCCCGAACACT
LM17801	54	F: TGTCCGAGTTGCTATGCT<br>R: ACCCACGAATGTTTCACTA
LM29637	54	F: TGATGCCTGCAAACCTTG<br>R: GACTGCCACTCTGCTTCCT
LM5948	54	F: CCTGCACTTGTGGTTGTT<br>R: CGACCGATCCCTTATTTG
LM12508	54	F: TCACGGCATACAACAAGG<br>R: TATCCACCGACCACTCAA
LM2253	58	F: GGCTACTGTACTTTGCGGTTAA<br>R: GGAGGAGGAATGGCTGGT
LM7247	56	F: GTCCTCTTCTGCCGATTC<br>R: GCTCTGATTTATTCTCCGATTT
LM28383	54	F: CCCTTTCCATTTACCACG<br>R: TCAGCGACTCCAGCCATC
LM24674	54	F: GTGGTGCGGTCTGGATGA<br>R: TCTGTTTCTCGGCAAGGT
LM6183	58	F: AGCCGGTGGTGACGATGT<br>R: CCCTTGACGAGGTAGAGGATAA
LM10081	60	F: TTTCTGCACCGTCAGTGGG<br>R: GCAACATCAGCGGCTTCAC
LM6856	54	F: CCATGTTACACCGTCCTA<br>R: CTCCAAGTCTGGTCCCTA
LM11271	52	F: GATACATGATTCGAGGGATT<br>R: AGTCAAGCGTCAAACAAC
LM15060	56	F: TGTTCCAGGTTCGACTTATCT<br>R: TGTCGCCACTATCCTTTCA
LM33865	52	F: GCTAGTAAATCGGAGGAC<br>R: TAGCCATAACACCAATCC
